# Porous Materials for Hydrolytic Dehydrogenation of Ammonia Borane

**DOI:** 10.3390/ma8074512

**Published:** 2015-07-21

**Authors:** Tetsuo Umegaki, Qiang Xu, Yoshiyuki Kojima

**Affiliations:** 1 Department of Materials & Applied Chemistry, College of Science & Engineering, Nihon University, 1-8-14 Kanda-Surugadai, Chiyoda-Ku, Tokyo 101-8308, Japan; E-Mail: kojima.yoshiyuki@nihon-u.ac.jp; 2 National Institute of Advanced Industrial Science and Technology (AIST), 1-8-31 Midorigaoka, Ikeda, Osaka 563-8577, Japan; E-Mail: q.xu@aist.go.jp

**Keywords:** hydrolysis of ammonia borane, porous materials, support, immobilization, nanostructured materials

## Abstract

Hydrogen storage is still one of the most significant issues hindering the development of a “hydrogen energy economy”. Ammonia borane is notable for its high hydrogen densities. For the material, one of the main challenges is to release efficiently the maximum amount of the stored hydrogen. Hydrolysis reaction is a promising process by which hydrogen can be easily generated from this compound. High purity hydrogen from this compound can be evolved in the presence of solid acid or metal based catalyst. The reaction performance depends on the morphology and/or structure of these materials. In this review, we survey the research on nanostructured materials, especially porous materials for hydrogen generation from hydrolysis of ammonia borane.

## 1. Introduction

Sustainable energy is a priority and one of the most essential requirements on the global agenda in the 21st century. Clean, effective, affordable and reliable energy is indispensable for global advancement and prosperity. The present energy system is based on the irreversible consumption of fossil resources. Due to their limited availability and environmental issues connected to their combustion, renewable sources of energy have drawn increasing attention in recent years. Seeking new and alternative energy sources has been one of the most difficult tasks because of the rise in prices to develop the energy sources, demands for fossil fuels, depletion or reduction of natural resources worldwide and fossil fuel reserves. Energy from many renewable sources, such as solar or wind power, cannot be gained on-demand and power is only supplied at specific times. For mobile applications, the energy has to be storable and transportable in a manner that can be handled easily and is efficient in terms of weight and volume. Faced with such obstacles, ways of storing energy from renewable sources become very interesting. Hydrogen has many advantages as an alternative energy carrier due to high energy density, and environmental friendliness [1,2,3,4,5,6,7,8]. The storage of electricity in the form of hydrogen suffers from penalties, especially from its low volumetric energy density. Production, storage and distribution of hydrogen from cheap and renewable sources with development of smart materials by using new technologies are the key factors for hydrogen energy utilization in real life. Hydrogen can be produced from chemical reactions [9], mainly from the reforming and thermal decomposition of hydrocarbons [10] in forms of liquid fuels (methanol, ethanol, gasoline, *etc.*). In this case, however, high-temperature reforming processes are too complex to satisfy the requirements of portable use. Alternative storage using chemical hydrides in the form of the liquid fuels could be employed as hydrogen sources. Among the chemical hydrides, ammonia borane is the most convenient material for a storage and supply system due to its advantages, such as high gravimetric/volumetric hydrogen storage capacity compared to other metal hydrides [11,12,13,14,15,16,17,18,19,20]. The compound has low molecular weight (30.9 g mol^−1^) and high hydrogen content of 19.6 wt. % or 140 g L^−1^. Moreover, ammonia borane has a high solubility in water (33.6 g per 100 mL) at room temperature and is stable in aqueous solution at room temperature [21,22,23,24,25,26,27,28,29]. Additionally, its non-toxic nature, non-flammability in basic aqueous solution, high stability in air, and the ability to control the hydrogen production can provide additional advantages. Moreover, the reaction co-product NH_4_BO_2_ can be recycled via an irreversible chemical reaction, in conjunction with off-board spent fuel regeneration [30], and hydrogen generation even at low temperatures can be possible with exothermic hydrolysis reactions. Ideal ammonia borane hydrolysis occurs according to the following reaction [31].

NH_3_BH_3_+ 2H_2_O → NH_4_^+^ + BO_2_^−^ + 3H_2_ (1)

The hydrolysis reaction can be accelerated and controlled by the use of a suitable catalyst including noble or non-noble transition metals/their alloys or salts besides acid accelerators [32] from ammonia borane solution [33,34,35,36,37,38,39,40,41,42,43,44,45,46,47,48]. The catalyst activity is highly dependent on the metal species, particle size, crystal structure, catalyst precursor and support materials employed [16]. Preparation of a porous catalyst with large surface area can enhance the catalytic activity [49]. Porous materials have attracted great attention and undergone rapid development due to their adjustable pore structure and multifunctionality that can be achieved by tailoring the composition, structure, chemical composition, surface properties, and ability to adsorb/absorb [50].

In this review, we briefly survey the research progress in porous materials for hydrolysis of ammonia borane.

## 2. Porous Support Materials

Support materials play not merely the role of carrier for active metal, but also provide a large active surface area and better dispersion of the active phase due to their porous nature. Besides that, a supported catalyst facilitates the diffusion of reactants through the pores to the active phase, which is the major limiting step for the hydrolysis reaction, improves the dissipation of the reaction heat, retards the sintering of the active phase, and increases the poison resistance. In comparison with various metals powder or their salt catalysts, the supported catalysts are highly appreciated in practical applications owing to their easy separation from fuel solution, and consequently, the ready controllability of the hydrolysis reaction and reusability of the catalyst [51,52]. In this section, we briefly survey porous support materials for supporting and immobilizing active species for hydrolysis of ammonia borane.

### 2.1. Microporous and Mesoporous Inorganic Support Materials

Porous materials are classified as macro-, meso- and microporous depending on the size of the pores, e.g., >50 nm, 50–2 nm and <2 nm, respectively. The use of microporous and mesoporous materials with ordered porous structures as the hosts to encapsulate metal nanoclusters has attracted particular interest in catalysis because the pore size restriction could limit the growth of nanoclusters and lead to an increase in the percentage of the catalytically active surface atoms. The use of nanocluster catalysts in systems with confined void spaces such as inside mesoporous and microporous solids appears to be an efficient way of preventing aggregation [53,54,55].

Co and Co borides/phosphides show attractive catalytic activities [56,57]. The Co–B catalyst is supported on highly ordered mesoporous silica particles prepared by chemical impregnation-reduction method with pore size of 2–10 nm [58]. Co–B nanoparticles on the mesoporous silica are able to block large numbers of mesopores by either completely filling the pores from inside or positioning on the pore face, while Co–B particles supported on non-porous silica are composed of spherical particles in the range of 30–40 nm, all or part of which are present in agglomerated state. In contrast, mesoporous silica particle-supported Co–B affords a large amount of particles (~90%) having a size lower than 10 nm. Co–B nanoparticles are located on the surface of the mesoporous silica particles with some portion of the former particles anchored into the pores. Co–B catalyst supported on mesoporous silica particles was able to produce the expected amount of hydrogen (H_2_/NH_3_BH_3_ = 3.0) by hydrolysis of ammonia borane, while unsupported Co–B catalyst and that which is supported on non-porous silica particles were able to produce only ~85% of H_2_ yield (H_2_/NH_3_BH_3_ = 2.55). Hydrolysis in the presence of unsupported powder and Co–B catalyst supported on non-porous silica particles is in the first order with respect to concentration of ammonia borane. By comparison, the hydrogen generation data produced by the Co–B catalyst supported on mesoporous silica particles powder was zero order with respect to ammonia borane concentration due to its high effective surface area that permits immediate hydrogen generation through surface reaction. The maximum hydrogen generation rate achieved with mesoporous silica particle-supported Co–B is about 2.5 and 3 times higher than that obtained with non-porous silica particle-supported Co–B and unsupported Co–B powder catalyst.

**Figure 1:**
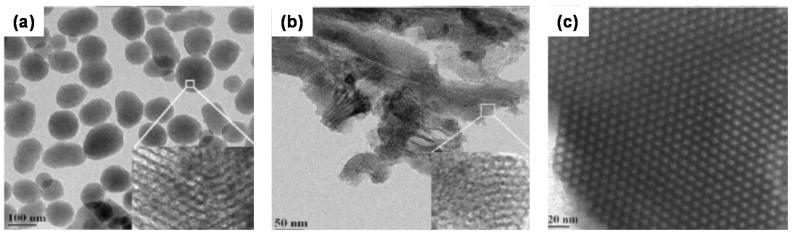
TEM micrograph of MCM-41 (**a**); FSM-16 (**b**); and SBA-15 (**c**). Reproduced with permission of Ref. [55].

The dispersion of the catalyst species depends on the types of mesoporous silica [55] (Figure 1). Co–B particles are located inside the pores of SBA-15 silica by keeping the pore structure intact while for MCM-41 and FSM-16 catalyst, particles either completely fill the pores or lie outside on the face of pores. The Co–B particles are well confined in the pores of SBA-15 acquiring the size of pores (~6 nm). Along the channel, the size of Co–B slightly increases to around 10 nm. In case of FSM-16 and MCM-41, the Co–B particles are well dispersed on the surface having broad distribution of size in the range from 3–30 nm, most of which (90%) have a size lower than 15 nm. A particle size greater than the pore size confirms that Co–B particles are located on the surface of the MCM-41 and FSM-16 type silica with some portion of the particle anchored into the pores. During the reduction process by sodium borohydride, the Co–B particles are formed by release of hydrogen gas. Due to the interconnected pore assembly, hydrogen can leave the interior of the SBA-15 easily. In FSM-16 and MCM-41, the pores are not connected and thus, hydrogen can be released only from the pore face which is blocked by the Co–B particles. Thus, due to the pressure exerted by the hydrogen gas, the Co–B particles are pushed out on the external surface of MCM-41 and FSM-16. The hydrogen production data for Co–B supported on all the mesoporous silica materials proves zero order kinetics with respect to concentration of ammonia borane. The maximum hydrogen generation rate achieved by Co–B supported on SBA-15 silica (~1900 mL-H_2_ g-(Co–B cat.)^−1^ min^−1^) is 4.2 and 5.3 times higher than that obtained by non-porous silica supported Co–B (~480 mL-H_2_ g-(Co–B cat.)^−1^ min^−1^) and unsupported Co–B powder catalyst (~360 mL-H_2_ g-(Co–B cat.)^−1^ min^−1^). For mesoporous silica supports, Co–B supported on SBA-15 showed the highest hydrogen generation rate which is about 1.5 times higher than that measured with MCM-41 (~1150 mL-H_2_ g-(Co–B cat.)^−1^ min^−1^) and FSM-16 (~1200 mL-H_2_ g-(Co–B cat.)^−1^ min^−1^). The activation energies of Co–B catalyst supported on SBA-15 (43 kJ mol^−1^) displays significantly lower energy barriers in comparison to Co–B supported on MCM-41 (51 kJ mol^−1^) and FSM-16 (58 kJ mol^−1^).

Co–B catalysts were also synthesized by pulsed laser deposition (PLD) in form of nanoparticle-assembled films because of easily controllable surface morphology and structure by the use of this method [22,59,60]. Nanoparticle-assembled Co–B thin film on a planar glass substrate was able to produce almost the expected amount of hydrogen (95%) from hydrolysis of ammonia borane with significantly higher rate (about six times) than the same amount of the corresponding Co–B powders [60]. The Co–B nanoparticles were produced during the ablation process on the film surface, with an average size of around ~250 nm and well established spherical form. Highly irregular and porous carbon film was adopted as a support for the Co–B nanoparticles to effectively improve the initial surface area and obtain better dispersion of nanoparticles. The films ranging from diamond-like to highly porous, cluster-assembled structures were deposited using PLD by varying laser parameters [61]. The total amount of hydrogen generated by hydrolysis of ammonia borane using both the Co–B films (H_2_/NH_3_BH_3_ = 2.85) is closer to the quantitative yield expected from the reaction stoichiometry (H_2_/NH_3_BH_3_ = 3.0) than that generated by Co–B powder (H_2_/NH_3_BH_3_= 2.55). The maximum hydrogen generation rate obtained for the carbon-supported Co–B films (~4060 mL-H_2_ g-cat.^−1^ min^−1^) has been found to be significantly higher than that obtained using the unsupported Co–B film (~2400 mL-H_2_ g-cat.^−1^ min^−1^) [62]. Both unsupported and supported Co–B catalyst films synthesized by PLD showed amorphous spherical particle-like morphology (average size ranging between 50 and 300 nm) with some agglomerates. The supported Co–B film had a dendritic microstructure with the Co–B nanoparticles embedded in the porous carbon film with improved dispersion. By comparison, the carbon film deposited at low Ar pressure exhibited a columnar structure with embedded spherical nodules on the surface. By increasing the pressure, dendritic, highly porous microstructure starts to appear with extremely irregular surface features which appear bigger, more loosely packed, and non-spherical with barely any adhesion to the substrate because cluster–cluster collision may also occur. Film-substrate adhesion is slightly poorer than that reached at low Ar pressure. Co–B catalysts supported on the carbon film deposited at low Ar pressures show almost similar catalytic activity as unsupported Co–B film due to the non-porous structure of the carbon films. However, the catalytic activity increases for Co–B catalysts supported on the carbon films deposited at higher Ar pressures. Hydrogen generation rate reached the maximum for the carbon film deposited at low pressure (40 Pa), and then, Co–B catalyst supported on the carbon film deposited at highest pressure of 50 Pa showed drastic decrease in the hydrogen generation rate and was not able to complete the hydrolysis reaction of ammonia borane due to its very weak adhesion with the substrate that leads to slow detachment of the film from the substrate in the reactant solution. The activation energy of the carbon film supported Co–B film is 29 kJ mol^−1^ which is lower than that obtained with unsupported Co–B film (34 kJ mol^−1^) and Co–B powder (44 kJ mol^−1^) [62].

Ni foam-supported amorphous ternary catalysts such as Co–Mo–B and Co–W–B have been prepared by a modified electroless plating method and exhibited enhanced hydrogen generation kinetics [58,63]. In a typical electroless deposition case, hydrogen evolution as a by-product is deliberately inhibited in order to produce a uniform and dense metallic coating by using strong complex agents and stabilizers in the bath solution. In the modified method, the formation of gas by increasing the concentrations of reducing agent and main salts, as well as elimination of the use of stabilizer was favored, and then, the nucleation and deposition rates of the metals are greatly increased, and a large number of hydrogen bubbles are evolved. These hydrogen bubbles function as a dynamic template and metal is chemically deposited and grows within the interstitial spaces between the hydrogen bubbles forming a porous coating of metallic particles on the support. Quaternary cobalt-tungsten-boron-phosphorus porous particles supported on Ni foam (Co–W–B–P/Ni), which are prepared through ultrasonification-assisted electroless deposition route, consisted of interconnected flower-like porous nanospheres (diameters: 200–400 nm) with enhanced contact between the active component and the substrate [64]. The molar ratio of generated hydrogen to the initial ammonia borane was close to 3.0, and the required reaction time with Co–W–B–P/Ni was much shorter than that with Co–W–B/Ni and Co–B/Ni. The catalytic hydrolysis reaction with respect to ammonia borane concentration exhibited quasi first-order character. Hydrogen generation rate in the presence of the most active catalyst was 4000 mL-H_2_ g-cat.^−1^ min^−1^. Moreover, the apparent activation energy for the quaternary catalyst was determined to be 29.0 kJ mol^−1^. After the 10th usage, the catalyst preserved 68% of its original hydrogen generation rate. The deposited catalyst layer did not peel off from the Ni foam substrate after repeated testing.

The use of zeolites as host materials with confined void spaces for guest metal nanoparticles seems to be one of the up-and-coming strategies to prevent the agglomeration of metal nanoparticles and bulk metal formation. Furthermore, encapsulation of metal nanoparticles within the porous structure of zeolite or between the zeolite-supported layers can help in the kinetic control of catalytic reactions [65].

**Figure 2:**
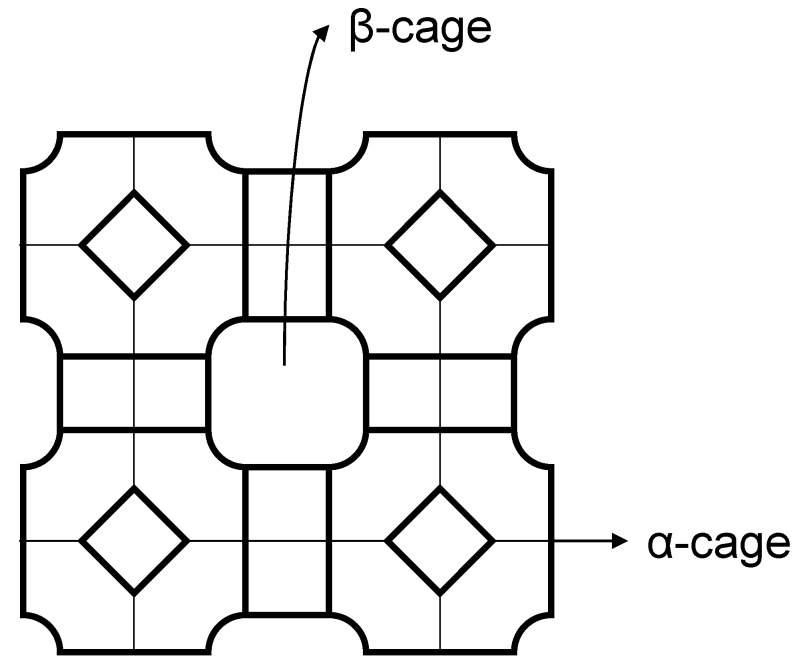
The framework structure of ZK-4 zeolite with α- and β-cage [66].

Ru nanoparticles@ZK-4 were prepared by ion-exchange of Ru^3+^ ions with the extra framework Na^+^ cations of ZK-4 zeolite (Na_9_[(AlO_2_)_9_(SiO_2_)_15_]·27H_2_O), LTA type structure (Linde-type A, Figure 2) with highly ordered cavities in 3-D structure, following sodium borohydride reduction of ruthenium(III)-exchanged ZK-4 in water at room temperature [66]. Neither the crystallinity nor the lattice of ZK-4 zeolite is altered by ion exchange. The incorporation of ruthenium(III) ions into ZK-4 zeolite and the reduction of ruthenium(III) ion forming Ru nanoparticles@ZK-4 causes no observable alteration in the framework lattice and no loss in the crystallinity of ZK-4 zeolite. Well dispersed ruthenium metal nanoparticles were present on the external surface of zeolite. The particle size of ruthenium nanoparticles was found to be in the range of 2.0–3.7 nm with a mean diameter of 2.9 nm. On passing from ZK-4 zeolite to ZK-4 zeolite and Ru nanoparticles@ZK-4, a notable decrease in the micropore volume and micropore area are observed, indicating that ruthenium nanoparticles exist not only on the surface but also inside the cavities of ZK-4 zeolite. The initial rates of hydrogen generation from the hydrolysis of ammonia borane are the highest using Ru nanoparticles@ZK-4 containing 1.1 wt. % Ru, mostly on the surface and readily accessible. They provide the complete stoichiometric hydrogen generation ([H_2_]/[H_3_NBH_3_] = 3) at 293 K. The activation energy was 28.2 kJ mol^−1^ and the turnover frequency (TOF) of Ru nanoparticles@ZK-4 was 5400 mol-H_2_ mol-Ru^−1^ h^−1^. The complete release of hydrogen is achieved in each of the successive catalytic runs (5 runs) without leaching of ruthenium into the reaction solution. The decrease (<15%) in catalytic activity in subsequent runs can be attributed to the passivation of active nanoparticle surfaces by boron products, e.g., metaborate, which decreases accessibility of active sites as previously seen in the case of Rh(0) nanoparticles@zeolite [36] and Cu(0) nanoparticles@zeolite [65].

Three-dimensional graphene-based framework (3DGF) like foams, sponges, and aerogels consist of exfoliated graphene sheets with lateral dimensions of several micrometers and an interconnected framework of ultrathin graphene nanosheets with porous structure (pore size: from a few hundred nanometers to several micrometers). Three-dimensional nitrogen-doped graphene-based frameworks (3D-(N)GFs) were synthesized using dicyandiamide as the nitrogen precursor, via a hydrothermal reaction and freeze-drying process. 3D-(N)GFs consist of three types of nitrogen: pyridinic nitrogen N (N1), pyrrolic nitrogen N (N2), and graphitic nitrogen N (N3). From the Raman spectrum, the slight upshift of the D and G bands of 3D-(N)GFs were observed because of the structural distortion of graphene induced by the different bond distances of C–C and C–N after doping. Ni^2+^ was introduced to the 3D-(N)GFs and subsequently reduced with sodium borohydride to obtain metallic nickel nanoparticles immobilized in three-dimensional nitrogen-doped graphene-based frameworks (NiNPs@3D-(N)GFs) [67]. Nanoparticles (2–4 nm) dispersed on the surface of the 3D-(N)GFs were obtained. At a relatively low pressure, appreciable decreases in nitrogen adsorption were observed for the Ni-loaded three-dimensional nitrogen-doped graphene based frameworks when compared with pristine graphene oxide, which can be interpreted by the existence of nickel nanoparticles on the graphene surface. With an increasing nitrogen content, the catalytic performance for hydrolysis of ammonia borane increases, and NiNPs@3D-(N)GFs with nitrogen content of 3.7% shows the highest activity. The incorporation of nitrogen strengthens the metal-support interactions and the effective immobilization of metal nanoparticles. Moreover, nitrogen doping leads to an increase in the surface chemical activity in terms of polarity and basicity. The activity in terms of TOF is 41.7 mol-H_2_ mol-Ni^−1^ min^−1^ for the as-synthesized NiNPs@3D-(N)GFs. The catalyst also shows good stability for recycle tests. The activation process occurs on the metal catalyst surface, as suggested by the zero order kinetics.

### 2.2. Polymer Gels for Support or Immobilization of Active Species

The design of advanced polymeric materials with controllable size, charge, functionality and porosity has great significance in obtaining efficient composites for use in hydrogen generation [68]. Nanocomposites containing polymer and inorganic particles have very good advantages for the preparation of artificial membranes with excellent separation performances, good thermal and chemical stability and adaptability to the harsh environments, as well as membrane forming ability [69,70,71,72].

Hydrogels are insoluble three-dimensional cross-linked hydrophilic polymeric materials. As the size of the hydrogel can be tuned from bulk to micro and/or nanometer, the pore size can also be designed as microporous, mesoporous and super porous up to a few micrometers [68]. Microgels are generally described as hydrogels with crosslinked polymeric particles below 100 nm that can be swollen by a good solvent. The high water content of microgels makes them soft and elastic with increased and tunable physical characteristics and these properties provide great resemblance to soft tissues [73,74]. Using silica precursors during microgel particle preparation allows composite material synthesis with adaptable surface and pore structure as silica particles are dissolvable via NaOH or HF treatments [75]. There has been an increased interest in using microgels as microreactors for templated synthesis of inorganic and/or metal nanoparticles [76]. Cryogels are sponge-like macroporous hydrophilic materials prepared by cryo-polymerization of monomers in the presence of ice crystals under frozen conditions [77,78]. Generally, cryogels are prepared by a free radical cryopolymerization reaction carried out at temperatures below 273 K, in the presence of a highly aqueous system containing monomers, cross-linker, and initiator. Upon the polymerization of the nonfrozen phase, 3-D gel formation occurs around the growing ice crystals which act as a template for the formation of the pores. Due to the crosslinking of polymer–polymer chains, a stable structure with macroporous gel network is revealed upon melting of the ice crystals at room temperature [79,80].

The use of cryogels in various catalytic reaction can offer additional advantages such as fast diffusion of the chemicals. Highly porous p(2-hydroxyethyl methacrylate) p(HEMA) cryogels were synthesized via cryopolymerization technique and used as templates for Co, Ni, and Cu nanoparticle preparation [81]. The size of metal nanoparticles within porous cryogel matrices was found to be 100–200 nm. The TOF values of p(HEMA)-Co, Ni and Cu were 3.8, 0.8 and 1.1 mol H_2_ mol-cat.^−1^ min^−1^ for hydrolysis of ammonia borane and the activation energy of the most active p(HEMA)-Co was 37.0 kJ mol^−1^. In every use, 100% conversion was obtained, while activity gradually decreased from 100%–57.7% at the end of the fifth use. The metal nanoparticles within poly(3-sulfopropyl methacrylate) (p(SPM)) cryogels are evenly distributed with a size of about few hundred nm in p(SPM)-M (M: Co, Ni, and Cu) composite cryogels [82]. The pore sizes of p(SPM) cryogel are a few tens of micrometers. The hydrogen generation rates for Co, Ni, and Cu-loaded cryogels for the hydrolysis reaction of ammonia borane were 2469, 1591, and 700 mL-H_2_ g-metal^−1^ min^−1^, respectively. The activation energies for hydrolysis of ammonia borane catalyzed by poly(vinyl imidazole)-Co capsule particles were reported as 51.6 kJ mol^−1^ [83], and 25.6 kJ mol^−1^ for p(SPM)-Co cryogel composite systems.

Poly(vinyl phosphonic acid) (p(VPA)) microparticle and composite p(VPA)-silica microparticle hydrogels are synthesized using a micro-emulsion polymerization technique. P(VPA) hydrogel with macro dimensions were shown for use as template in the preparation of metal nanoparticles such as Co, Ni, and Cu [84,85]. The preparation of p(VPA) in a smaller dimension can offer better catalytic performance for their *in situ* prepared metal nanoparticles and tunable porosities by inclusion of silica moieties. P(VPA) microgels synthesized with *N*,*N*′-methylenebisacrylamide (MBA) crosslinker are spherical in shape. The particle sizes vary from a few hundred nm to 20 mm. To generate additional pores in p(VPA) microgels, the prepared p(VPA)-Si composite was treated with 0.5 M NaOH to remove the silica particles. Metal nanoparticles, Co, Ni, and Cu are generated *in situ* inside these hydrogels by chemical reduction of the absorbed metal ions with a reducing agent such as sodium borohydride. The hydrogen generation rates for Co, Ni, and Cu-loaded microgels for hydrolysis of ammonia borane were 3272, 1538, and 433 mL-H_2_ g-metal^−1^ min^−1^, respectively. The activation energy for hydrolysis of ammonia borane catalyzed by p(VPA)-Co microgels was 25.5 kJ mol^−1^. The conversion was 100% until the end of the third use, and reduced to 90% at the end of the 10th use. The catalytic activity of bulk p(VPA)-Co is significantly reduced to 15% after the fifth use, whereas, p(VPA)-Co microgel has very good catalytic activity and performance. Hydrogen generation rates for porous p(VPA)-Co and p(VPA)-Co microgels were 2033 and 1407 mL-H_2_ g-Co^−1^ min^−1^, respectively. Accordingly, the porosity in the microgel can generate high metal ion loading capacity, and fast hydrogen production rates. P(VPA) microgels have pore sizes of 17.4 nm, whereas p(VPA)-Si and porous p(VPA) microgels have 25.1 and 60.2 nm pore sizes, respectively.

### 2.3. Metal-Organic Frameworks for Immobilization of Active Species

Metal-organic frameworks (MOFs) synthesized by assembling metal ions with organic ligands have recently emerged as a new class of porous materials for their amenability to design as well as fine-tunable and uniform pore structures. Their distinct characteristics make them very promising for a variety of applications, including gas storage and separation, sensing, optics, drug delivery, and catalysis [86,87,88,89,90,91,92,93,94,95,96,97]. By serving as unique host matrices, the potential applications of MOFs can be extended further by encapsulating metal nanoparticles within the frameworks. MOFs have been utilized as supports for metal nanoparticles since they provide a powerful confinement effect to limit the growth of metal nanoparticles. However, the precursor compounds and products can actually diffuse out through the pores of the host to form the metal nanoparticles with aggregation on the external surface of MOFs. To circumvent these drawbacks, great efforts have been made.

The MOF compound, Co_2_(bdc)_2_(dabco) (bdc = 1,4-benzenedicarboxylate; dabco = 1,4-diazabicyclo[2.2.2] octane), is used as the precursor to synthesize a catalyst with dispersed Co(0) metal sites [98]. The catalytically effective Co(0) sites are stabilized by the organic molecules, which are used to coordinate to Co (II) in the MOF precursor. The precursor of Co-MOF has a 3-D pillared structure with an accompanying 3-D pore system. When ammonia borane is added to a mixture of Co-MOF and sodium borohydride, the framework of Co-MOF collapses and the amorphous Co(0) catalyst is formed *in situ* through the reduction of the Co-MOF. The resulting catalytically active Co(0) sites are separated by the residue of the precursor of Co-MOF and stabilized by the organic linkers. A hydrogen generation rate of 932,000 mL-H_2_ mol-cat^−1^ min^−1^ was obtained in 0.32 M ammonia borane. Co(OH)_2_ is formed during the reaction and deposited on the surface of the catalyst when it is exposed to air, resulting in a significant decrease in catalytic activity.

ZIF-8 was demonstrated to have a strong ability in immobilizing Ni nanoparticles, preventing their aggregation and therefore increasing their catalytic surface area [99]. Highly dispersed Ni NPs immobilized on ZIF-8 (Ni/ZIF-8) have been prepared and their catalytic activity studied for hydrolysis of ammonia borane. A small volatile molecule nickelocene Ni(cp)_2_ was used as a precursor of Ni nanoparticles. ZIF-8 incorporating Ni(cp)_2_ [Ni(cp)_2_/ZIF-8] were synthesized via chemical vapor deposition and chemical liquid deposition and subjected to reduction by H_2_/Ar at 573 K with different Ni loadings. The most active sample was a CVD-Ni/ZIF-8 sample in which Ni nanoparticles were highly dispersed on ZIF-8 and exhibited the smallest size (2.7 nm). The TOF value was 14.2 mol H_2_ mol-Ni^−1^ min^−1^ at room temperature. No significant decrease in catalytic activity was observed even after five consecutive hydrolysis reactions.

**Figure 3:**
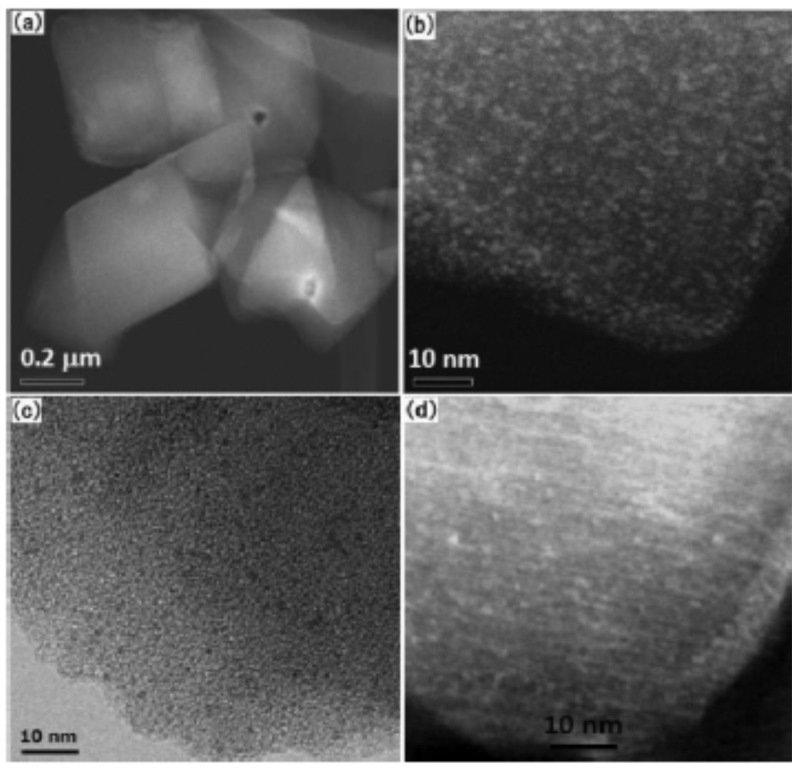
(**a**,**b**) HAADF-STEM; (**c**) TEM images; and (**d**) reconstructed slice by tomography of 2 wt. % Pt@MIL-101. Reproduced with permission of Ref. [46]. Copyright 2012 American Chemical Society.

Ultrafine metallic Pt nanoparticles have been supported onto MIL-101(Cr) without aggregation on the external surface (Figure 3) and their catalytic activity has been investigated [46]. Pt@MIL-101(Cr) with Pt loadings up to 5 wt. % exhibits the same crystallinity as the parent MIL-101(Cr), indicating that the integrity of the MIL-101 framework was maintained during the preparation procedure. 2 wt. % Pt@MIL-101(Cr) exhibited a uniform distribution of Pt nanoparticles of 1.2–3 nm (average 1.8 nm) in the MIL-101(Cr) framework. The Pt@MIL-101(Cr) required 2.5 min to complete the hydrolysis of ammonia borane which corresponds to ~1.0 × 10^7^ mL-H_2_ mol-Pt^−1^ min^−1^ at room temperature. Pt@MIL-101(Cr) was reused for five runs with no significant drop in its activity and no changes in the MIL-101(Cr) framework or in the morphology of Pt nanoparticles was observed.

Alloying a parent metal with a second metal offers numerous opportunities for modulating the electronic structures of catalysts and optimizing their catalytic performance [100,101]. When the noble metal precursors are loaded, they can be treated by the hydrogen and plasma reduction methods under relatively moderate conditions which are suited perfectly for the preparation of noble nanoparticles hosted inside the MOFs [46], but not for non-noble metal based nanoparticles because of contradictions between the high reduction temperatures of non-noble metals and the low thermal stabilities of MOFs. Therefore, a general and facile method that can easily control the nucleation and growth of metal nanoparticles, especially non-noble metal-based nanoparticles, with high uniformity only inside the pores of MOF is still imperative. A double solvents method introduced noble metal precursors into the MOF pores without metal nanoparticle aggregation on the external framework surface after the precursors underwent hydrogen reduction at a relatively low temperature [46]. However, the hydrogen reduction method is not suited for non-noble metal precursors because of contradictions between the high reduction temperatures of non-noble metals and the limited thermal stabilities of MOFs. To circumvent this drawback, an overwhelming reduction approach in solution at room temperature were exploited which could encapsulate the non-noble metal nanoparticles within the MOF pores [102].

AuNi alloy nanoparticles have been immobilized onto the pores of MIL-101(Cr), a chromium-based MOF with molecular formula Cr_3_F(H_2_O)_2_O[(O_2_C)C_6_H_4_(CO_2_)]_3_·*n*H_2_O (where *n* = ~25) [103]. MIL-101 has incredibly large pore size (2.9–3.4 nm) and high specific surface area (5900 m^2^ g^−1^). The pore windows with diameters of ~1.2 and 1.6 nm are big enough for the precursor compounds HAuCl_4_ and NiCl_2_ to diffuse into the pores, within which nucleation can take place to form the AuNi alloy nanoparticles. Because of the large inner surface area of MIL-101 with hydrophilic character derived from the metal-cluster based trimeric building block, the small amount of aqueous precursor solution, with a volume slightly less than the pore volume of the adsorbent, was readily incorporated into the pores of dehydrated MIL-101, which was suspended in a large amount of dry n-hexane, by capillary force. After loading the precursors and drying the metal precursor/MOF composite, an overwhelming reduction approach with a high-concentration of sodium borohydride solution was carried out for avoiding metal nanoparticle aggregation on external surfaces of MIL-101 framework. When a high-concentration of sodium borohydride solution (0.6 M) is used, highly dispersed AuNi alloy nanoparticles with average size of 1.8 nm were encapsulated within the pores of MIL-101 without deposition on the external surface. No large particle aggregation and uniform 3D distribution of monodispersed AuNi nanoparticles throughout the interior cavities of MIL-101 crystals was observed. After the impregnation and reduction processes, there is no loss of the crystallinity for AuNi@MIL-101, indicating that the integrity of the MIL-101 framework is maintained. The appreciable decreases in the surface areas and the pore volumes of M@MIL-101 indicate that the pores of the host frameworks are occupied by dispersed metal nanoparticles and/or blocked by the metal nanoparticles located on the surface. The AuNi@MIL-101 catalysts are more active for the hydrolysis of ammonia borane than the monometallic counterparts, exhibiting a synergistic effect between Au and Ni. The AuNi@MIL-101 with the Au/Ni atomic ratio of 7:93 shows a TOF of 66.2 mol-H_2_ mol-cat^−1^ min^−1^. The productivity of hydrogen over the AuNi@MIL-101 catalyst remained almost unchanged after five runs. Since the degenerative performance can be recovered totally after catalyst recycling, the slight activity drop should be attributed to the increase in concentration of metaborate and the viscosity of the solution during the hydrolysis of ammonia borane. Once metaborate generated during the reaction was removed from solution, the catalyst exhibited its original catalytic activity. No significant change in the morphologies of AuNi nanoparticles was observed along with retention of the MIL-101 framework were observed.

Ultrafine AuCo alloy nanoparticles were also encapsulated in the pores of MIL-101 without aggregation on the external surfaces of the host framework by using the double solvents method combined with the overwhelming reduction approach [103]. Highly dispersed and homogeneous AuCo alloy particles with size of *ca.* 1.8 nm were formed in the MIL-101 framework. The BET surface areas of MIL-101 and AuCo@MIL-101 are 3452 and 1930 m^2^ g^−1^, respectively. The AuCo@MIL-101 with the Au/Co atomic ratio of 6:94 is the most active with TOF value of 23.5 mol-H_2_ mol-cat^−1^ min^−1^. There was no significant decrease in catalytic activity even after five runs of hydrolysis reactions for the catalysts. No significant change in the morphologies of AuCo NPs were observed, along with retention of the MIL-101 framework.

Table 1 lists particle sizes and TOF of various catalysts discussed in this section. From this table, TOF of various catalysts appear to depend not only on the particle size, but also on other factors such as the particle dispersion, and metal-support interaction. Effective porous materials with high activity for hydrolytic dehydrogenation of ammonia borane are MOF, zeolite, and graphene nanosheet. Especially, uniform distribution of metal nanoparticles without aggregation after catalysis confirms the advantage of metal nanoparticles within the MOF matrix.

**Table 1:** Particle size of active species and turnover frequency (TOF) of various catalysts.

Catalysts	Active Species	Particle Size (nm)	TOF (mol-H_2_ min^−1^ mol-Active Species^−1^)	Ref.
Co–B/SBA-15	Co–B	6–12	3.4	[55]
Co–B/MCM-41	-	3–30	2	-
Co–B/FSM-16	-	3–30	2.1	-
Co–B/non-porous	-	30	0.9	-
unsupported Co–B	-	30–40	0.6	-
Co–B/C-film	-	50–300	0.6	[62]
unsupported Co–B film	-	~250	0.1	-
Co–W–P–B/Ni foam	-	200–400	-	[64]
Ru@ZK-4	Ru	2.9	90	[66]
Ni@3D-(N)GFs	Ni	2–4	41.7	[67]
p(HEMA)-Co	Co	-	3.8	[81]
p(HEMA)-Ni	Ni	-	0.8	-
p(HEMA)-Cu	Cu	-	1.1	-
p(SPM)-Co	Co	-	5.8	[82]
p(SPM)-Ni	Ni	-	3.8	-
p(SPM)-Cu	Cu	-	1.8	-
p(VPA)-Co	Co	-	7.7	[85]
p(VPA)-Ni	Ni	-	3.6	-
p(VPA)-Cu	Cu	-	1.1	-
Co-MOF	Co	<10	20.8	[98]
Ni-ZIF-8	Ni	2.7	14.2	[99]
Pt@MIL-101(Cr)	Pt	1.2–3	446.4	[46]
AuNi@MIL-101(Cr)	AuNi	2.9–3.4	66.2	[103]
AuCo@MIL101(Cr)	AuCo	1.8	23.5	[102]

## 3. Nanostructured Materials

An efficient approach to tune the reactivity of a catalytic material is nanostructuring. Nanodesign of materials, with hierarchical nanometric channels [104], containing both interconnected macroporous and mesoporous structures, is under investigation for a wide range of applications in different fields [105,106]. Nanostructured hollow spheres have attracted more and more attention in recent years, because of their special structures and unique properties [107]. For example, the void space in the hollow structure has been used to show the particle’s ability to withstand the cyclic change in volume [108], modulate refractive index [109], increase active area for catalysis and encapsulate sensitive materials (e.g., drugs, cosmetics and DNA) [110].

Ni_1−*x*_Pt*_x_* hollow spheres were synthesized through a template route by a replacement reaction using poly(styrene-*co*-methacrylic acid) (PSA) spheres (*ca*. 240 nm) as sacrificial templates [34]. Ni is first deposited on the surface of the PSA spheres and Ni^2+^ is reduced by sodium borohydride. The formation of Ni_1−*x*_Pt*_x_* is attained through the replacement reaction between K_2_PtCl_6_ and Ni, where the driving force comes from the large standard reduction potential gap between Ni^2+^/Ni and PtCl_6_^−^/Pt redox pairs. After the partial consumption of Ni, the resultant Ni_1−*x*_Pt*_x_*-PSA core-shell structures are dispersed in a toluene solution to dissolve the PSA template cores, resulting in the formation of Ni_1−*x*_Pt*_x_* metallic alloy hollow spheres. Pt and Ni atoms are distributed homogeneously and randomly at the Ni position. Both complete and broken hollow spheres are obtained. The wall thickness of the hollow sphere is 20–40 nm and the porous shell consists of smaller nanoparticles and nanowhiskers. The as-synthesized alloy hollow sphere catalysts, treated by filtering and washing, can be repeatedly utilized with no significant catalytic deactivation in the hydrolysis experiment. The activation energy of the hydrolysis of ammonia borane is determined to be 30 kJ mol^−1^ for Ni_0.88_Pt_0.12_, respectively. The amount of hydrogen generated by Ni_0.88_Pt_0.12_ hollow spheres increases nearly linearly with time before the hydrolysis reaction approaches its end, indicating an example of quasi zero-order kinetics.

Hollow nickel nanospheres with an average diameter of 170–450 nm and an average shell thickness of 55–60 nm were obtained by the solvothermal method at 423 K adjusting volume ratios of ethyl alcohol/ethylenediamine [111]. At the ratio of 4:6, the nickel hollow nanospheres are relatively uniform. When the ratio reaches 5:5, the uniformity of nickel hollow nanospheres becomes worse, and lots of large nickel particles were also formed. At the lower temperature of 393 K, uniform nickel spheres without obvious shells were obtained. While at a higher temperature of 453 K, besides the formation of Ni hollow spheres, aggregated Ni particles can also be formed and the average diameter of the nickel hollow spheres was also increased. The average size of the crystallized nanoparticles including the hollow spheres is 25–30 nm. Ni^2+^ cations in solution reacted with excess ethylenediamine to form a relatively stable structure of the [Ni(en)_3_]^2+^ complex, which cannot be reduced by sodium borohydride directly. The Ni/Pt hollow bimetallic nanospheres were also synthesized through the replacement reaction by using the as synthesized hollow nickel nanospheres as sacrificial templates. The average diameter and wall thickness of the Ni/Pt hollow bimetallic nanospheres are 250 nm and 40–55 nm, respectively. The rate of hydrogen generation on the molar ratio of 85:15 of Ni/Pt hollow bimetallic nanospheres reaches 5920 mL-H_2_ g-cat.^−1^ min^−1^.

Spherical particles of silica–nickel composite with hollow feature were obtained by sol-gel based method using PS templates [112,113,114,115]. There are two main peaks at around 300 and 680 nm in the particle size distribution profile of the hollow spheres prepared with Si + Ni content = 1.06 × 10^−3^ mol. The peak at around 300 nm in the profile of the hollow spheres prepared Si + Ni content = 3.19 − 10.64 × 10^−3^ mol is narrower and larger than that in the profile of the hollow spheres prepared with Si + Ni content = 1.06 × 10^−3^ mol, while the number of 680 nm particles drastically decreases with increase of Si + Ni content, indicating that homogeneity of the hollow silica-nickel composite spheres increases with the increase of Si + Ni content. The evolution of the molar ratios of the hydrolytically generated hydrogen to the initial ammonia borane, 2.3–2.4, was finished from 32–18 min in the presence of hollow silica-nickel composite spheres prepared with Si + Ni content = 1.06 − 10.64 × 10^−3^ mol. Clustered nickel species in hollow silica-nickel composite spheres increase with increase of Si + Ni content, and more reducible nickel species exist in the hollow spheres prepared with higher Si + Ni content. The stirring procedure in toluene is effective for reducing the amount of residual PS templates. The molar ratios of the hydrolytically generated hydrogen to the initial ammonia borane improved up to 2.6 after 22 min. The activation energies were 44–54 kJ mol^−1^ for the hollow spheres.

The wall thickness of hollow silica-zirconia composite spheres (*ca*. 200–300 nm diameter) was controlled by adjusting the amount of polystyrene template suspension from 2.0–17.5 nm [116]. The molar ratios of hydrolytically generated hydrogen to the initial ammonia borane in the presence of hollow silica-zirconia composite spheres with wall thickness from 17.5–2.0 nm are from 0.5–2.0. With decrease of wall thickness of the hollow spheres, both the specific surface area of the hollow spheres and the molar ratio of hydrolytically generated hydrogen to the initial ammonia borane in the presence of the hollow spheres increase. The molar ratio increases four times with the specific surface area increasing two times, indicating that the factors other than specific surface area can influence the catalytic activity of the hollow silica-zirconia composite spheres. The primary particles may form layer-like structures in the wall of hollow silica-zirconia composite spheres with the number of layers depending on the wall thickness.

Hollow silica-alumina composites were obtained by the same method using polystylene templates with and without calcination [117,118]. The calcination condition significantly influenced the morphology and activity for hydrolysis of ammonia borane, and homogeneity of the hollow spheres played an important role. Homogeneous hollow composite spheres were obtained upon calcination at 873 K. The molar ratios of hydrolytically generated hydrogen to the initial ammonia borane were 2.6 for 12 min, respectively. The amount of Brønsted acid sites of the hollow spheres had a significant impact on their activity.

**Figure 4:**
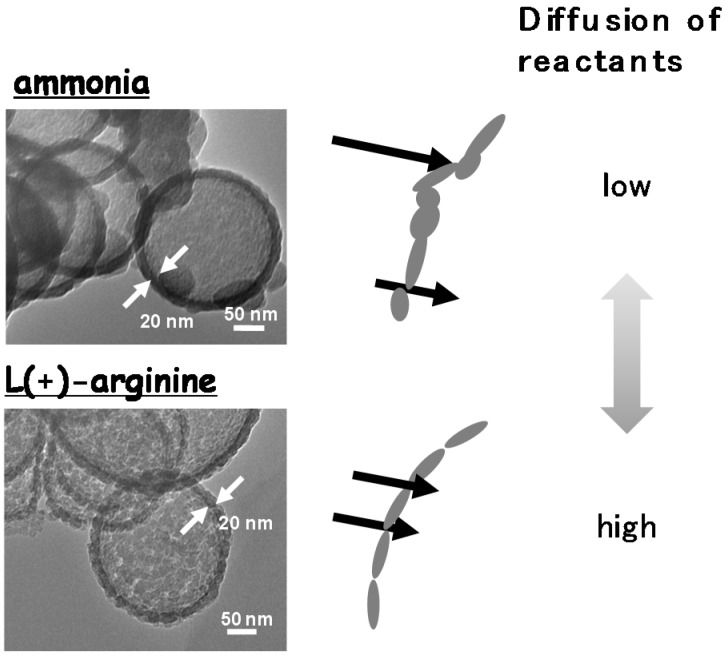
Difference in morphology of hollow silica-alumina composite spheres prepared using ammonia and L(+)-arginine [119].

Hollow silica-alumina composite spheres with diameters of 200–300 nm were also prepared by the same method using L(+)-arginine as a promoter of the sol-gel reaction [119]. The wall thickness of the samples prepared using L(+)-arginine and 7.5, 15.0, 30.0 g of PS template suspension were 50.0, 30.0, and 20.0 nm, respectively. The BET surface area and pore volume of the hollow spheres depend on the shell thickness; however, across all the samples, the average pore size was the same. Almost all the pores in the hollow spheres prepared using L(+)-arginine are micropores and mesopores, and a narrower pore size distribution is observed for the hollow spheres prepared using L(+)-arginine than in the hollow spheres prepared using conventional ammonia. The molar ratio of hydrolytically generated hydrogen to the initial ammonia borane in the presence of hollow spheres prepared using L(+)-arginine with wall thickness = 50.0, 30.0, 20.0 nm, and hollow spheres prepared using ammonia with wall thickness = 20 nm are 0.9, 2.4, 2.5, and 2.3 for 4, 5, 7, and 13 min, respectively. Dispersion of the primary particles of the hollow spheres prepared using L(+)-arginine is higher and/or the primary particles are well-ordered compared with the hollow spheres prepared using ammonia (Figure 4).

Porous nanowires have attracted a lot of attention because they present the advantages of both 1D nanostructures and high surface area materials. Co nanowires with highly ordered connected macroporosity were obtained by combining self-assembly of polystyrene spheres, a hard templating, together with electrodeposition [120]. The electrodeposition of Co inside the membrane channels followed by polycarbonate membrane and sphere elimination creates a close-packed array of aligned and monodispersed porous nanowires. The as-produced nanowires show a uniform radius (*r* = 500 nm), which matches that of the polycarbonate membrane channels. 3D ordered macropores are formed inside the submicron wire structures as the replication of the 100 nm radius ordered PS spheres. Smaller holes appeared to connect the macropores; these holes result from the contacts between the original polystyrene spheres. In addition, the macropores are all connected together with holes. In addition, the nanowire diameter (100–2500 nm), the macropore sizes and the surface areas can be tuned over a wide range using polycarbonate membranes and polystyrene spheres with different radii (35–500 nm). The nanowires totally hydrolyze ammonia borane within 90 min whereas with the nonporous nanowires the conversion reaches only 33%. Hydrogen generation rates of *ca*. 3.4 and 0.5 mL min^−1^ were calculated, respectively. With the decrease of diameter of PS spheres, the hydrogen generation rate decreased.

Table 2 lists TOF of various catalysts discussed in this section. From this table, metal based catalysts display relatively high activity. The activity depends on several factors such as compositions, morphology, and homogeneity of the nanostructured materials.

**Table 2:** TOF of various catalysts.

Catalysts	Active Species	TOF (mol-H_2_ min^−1^ mol-Active Species^−1^)	Ref.
Hollow Ni–Pt spheres	Ni-Pt bimetal	19.2	[111]
Hollow SiO_2_–Ni spheres	Ni	3.8	[114]
Hollow SiO_2_–Al_2_O_3_ spheres	SiO_2_–Al_2_O_3_	0.01	[119]
Co nanowire	Co	0.9	[120]

## 4. Conclusions

The present review briefly surveys porous materials for hydrolytic dehydrogenation of ammonia borane. We mainly discussed porous materials as support materials for active species and as nanostructured catalysts. These materials are effective not only in increasing the number of active sites by controlling particle size and improving the durability by confining and/or protecting the active species in micropores or mesopores, but also control of diffusion of reactants and/or by-products by controlling pore sizes. TOF of various catalysts appear to depend not only on the particle size, but also on other factors such as the particle dispersion, and metal–support interaction. Effective porous materials with high activity are MOF, zeolite, and graphene nanosheets because uniform distribution of metal nanoparticles without aggregation after catalysis confirms the advantage of metal nanoparticles within the matrix materials. Metal–support interactions between active metal species and porous materials play important roles not only in the catalytic activity but also in immobilization of active metal species. These results bring to light new opportunities in the development of high performance heterogeneous catalysts by using functionalized cavities of porous materials as hosts for ultrafine nanoparticles. Among nanostructured materials, metal based nanostructured catalysts display high activity. The activity depends on several factors such as compositions, morphology, homogeneity of their nanostructure, and so on.

Considering their practical use, it is required for these materials to increase their activity even in solution with a high concentration of reactants and/or by-products. The influence is probably reduced by controlling the diffusion rate of the reactants and/or by-products through pores of the catalyst materials. Controlling not only size of the materials’ pores but also fabricating well-ordered pores can also be important factors to control the diffusion rate of the reactants and/or by-products.
